# IL-1β induced HIF-1α inhibits the differentiation of human FOXP3^+^ T cells

**DOI:** 10.1038/s41598-017-00508-x

**Published:** 2017-03-28

**Authors:** Lea M. Feldhoff, Cesar M. Rueda, Maria E. Moreno-Fernandez, Johanna Sauer, Courtney M. Jackson, Claire A. Chougnet, Jan Rupp

**Affiliations:** 1 Department of Infectious Diseases and Microbiology, University of Lübeck, Lübeck, Germany; 2 Division of Immunobiology, Cincinnati Children’s Hospital Research Foundation, Department of Pediatrics, University of Cincinnati College of Medicine, Cincinnati, Ohio USA

## Abstract

Differentiation of regulatory Treg (Treg) in the periphery is critical to control inflammatory processes. Although polarization of inducible Treg (iTreg) often occurs in an inflammatory environment, the effects exerted by inflammation on human iTreg differentiation have not been extensively studied. We observed that IL-1β significantly reduced the frequency of FOXP3^+^ T cells under iTreg-polarizing conditions. Mechanistically, we show that IL-1β activated mTORC1 and downstream upregulated hypoxia inducible factor-1 (HIF-1α) expression. Using specific inhibitors, we demonstrated that both steps were critical in the deleterious effect of IL-1β on Treg differentiation. Chemical stabilization of HIF-1α by Dimethyloxalylglycine (DMOG) also significantly impaired iTreg differentiation. Interestingly, while IL-1β-treated cells exhibited only minor changes in metabolism, DMOG treatment decreased iTreg mitochondrial respiration and increased their glycolytic capacity. In conclusion, exposure to inflammatory stimuli profoundly inhibits human Treg differentiation HIF-1α dependent, suggesting that targeting HIF-1α could be a strategy to foster iTreg differentiation in an inflammatory milieu. However, IL-1β deleterious effect does not appear to be completely driven by metabolic changes. These data thus suggest that several mechanisms contribute to the regulation of iTreg differentiation, but the timing and respective requirement for each pathway vary depending on the milieu in which iTreg differentiate.

## Introduction

Regulatory T cells (Treg) control both innate and adaptive immune responses and are essential to prevent exacerbated inflammatory processes in pathological conditions^1, 2^. Treg originate from two distinct pathways, either through thymic development or by peripheral differentiation from conventional T cells (Tcons). Inducible Tregs (iTreg) can be differentiated *in vitro* from Tcons in presence of IL-2 and TGF-β^3–5^. Dendritic cell-mediated signals, such as indoleamine 2,3-dioxygenase or retinoic acid (RA), induce or stabilize iTreg conversion^6, 7^.

The environment in which iTreg differentiate could alter their differentiation. Notably, it is important to understand the effect of an inflammatory environment on this process. Inflammation, as hypoxia, is a potential inductor of the hypoxia inducible factor-1 (HIF-1) in various cell types, such as T cells^8–11^. HIF-1 consists of two subunits; HIF-1β is constitutively expressed, while HIF-1α is degraded by oxygen-dependent prolyl hydroxylation under normoxic conditions (21%)^12^. Besides hypoxia, HIF-1α is also stabilized in murine T cells by TCR stimulation and pro-inflammatory cytokines^10, 13, 14^. Transcription is an important mechanism of HIF-1α up-regulation in human CD4^+^ T cells, whereas post-translational regulation seems to play only a minor role^15^. HIF-1α acts as a metabolic rheostat in murine CD4^+^ T cells, notably boosting aerobic and anaerobic glycolysis^16, 17^. Further, in mice, HIF-1α promotes TH17 differentiation, while it inhibits iTreg differentiation^10, 18, 19^.

Importantly, the impact of inflammatory cytokines or metabolic changes on human iTreg differentiation is less studied. It is not clearly established whether HIF-1α is also a critical rheostat that integrates inflammatory and metabolic stimuli to abrogate human iTreg differentiation. Herein, we addressed these questions, by using the well-established model of iTreg differentiation *in vitro* ^20–23^, and determining the effect of IL-1β on this process. Additionally, we used chemical HIF-1α stabilization by Dimethyloxalylglycine (DMOG) to analyze the role of HIF-1α up-regulation in Treg differentiation.

## Results

### IL-1β abrogates iTreg differentiation

To determine how inflammation affects human iTreg differentiation, conventional T cells (Tcons, defined as CD4^+^CD25^−^CD127^high^ cells) were stimulated with anti-CD3/anti-CD28 beads in presence of TGF-β (1 ng/ml) and IL-2, in presence or absence of IL-1β. TCR stimulation led to IL-1β receptor (IL-1R) upregulation (Figure S1a), as previously reported^24, 25^. Relative to non-polarized, activated Tcons, iTreg polarization in absence of IL-1β led to approximately 40–60% of T cells expressing FOXP3 at d6, which is consistent with previous data^26, 27^. Higher concentration of TGF-β (2 ng/mL or 5 ng/mL) did not increase the frequency of FOXP3^+^ cells (Figure S1b); TGF-β in a concentration of 1 ng/ml was thus used throughout the study. Because iTreg produce less IL-2 compared to non-Treg^28, 29^, we confirmed that IL-2 production was mainly detected in FOXP3^−^ cells (Figure S1c).

**Figure 1:**
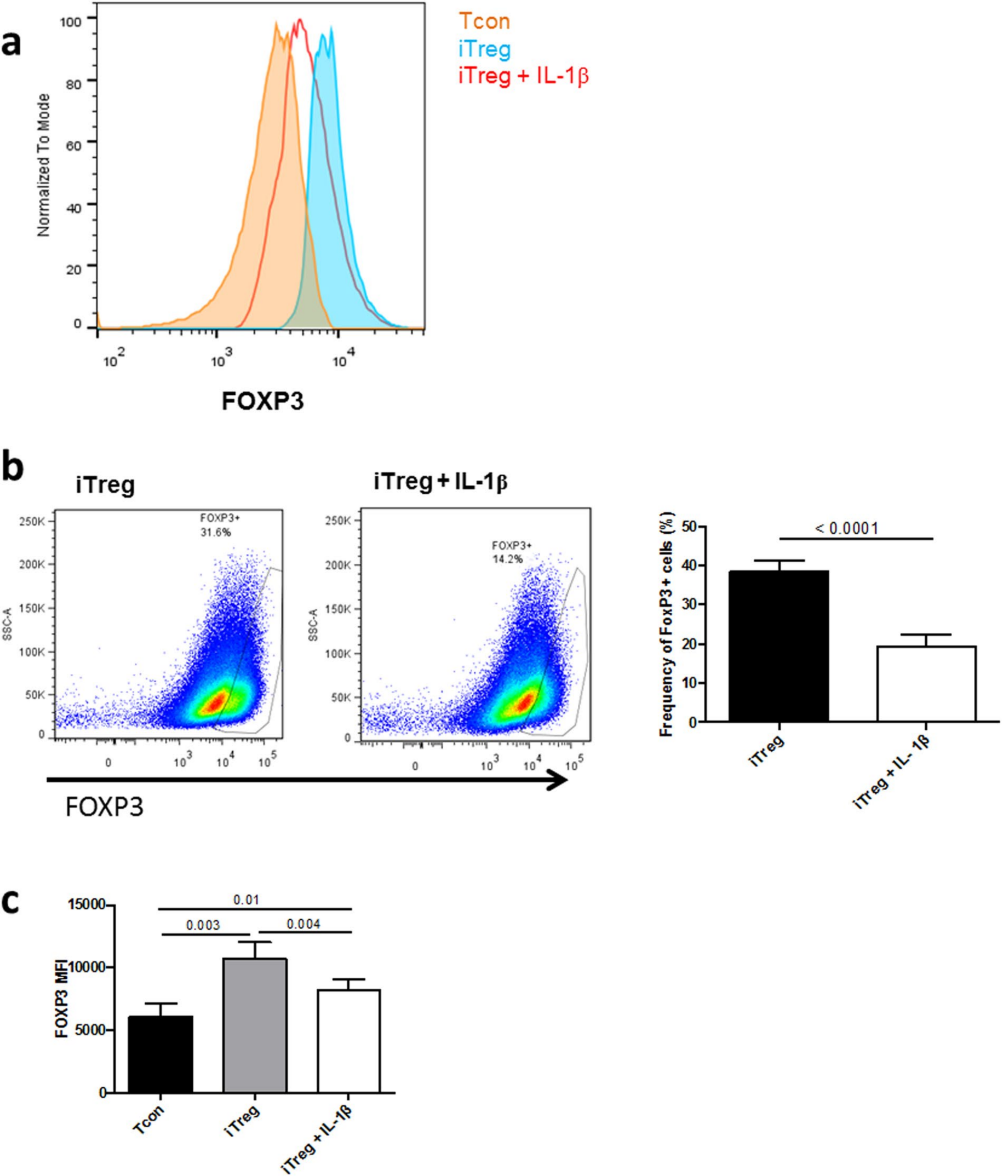
IL-1β abrogates iTreg differentiation. (**a**) Representative flow cytometry histogram showing FOXP3 expression in CD4^+^ cells, cultured for 6 days in unpolarized conditions (Tcon) or Treg-skewing conditions (iTreg), in absence or presence of IL-1β (100 ng/mL). (**b**) Left panel: representative FACS dot plots of FOXP3^+^cells of CD4^+^ cells at d6 of iTreg polarization with and without IL-1β stimulation. Right panel: mean (±SEM) percentage of FOXP3^+^ cells in iTreg cultured in presence or absence of IL-1β (n = 12). Threshold of positivity was set according to unpolarized Tcon cells. (**c**) Mean (±SEM) FOXP3 MFI in iTreg cultured in presence or absence of IL-1β (n = 12). P values correspond to paired t-tests.

Importantly, the presence of IL-1β during differentiation significantly decreased FOXP3^+^ cell frequency (Fig. 1a,b), as well as FOXP3 MFI (Mean fluorescence intensity) in the FOXP3^+^ subpopulation (Fig. 1c). Continuous exposure to IL-1β was required to inhibit FOXP3 induction, as a single IL-1β treatment at d1, or d3, did not change iTreg percentage (data not shown). Importantly, IL-1β did not impair cell viability and did not affect iTreg cell cycle entry, as evidenced by the similar LIVE/DEAD and Ki67 staining in treated and untreated cells (Figure S2).

### IL-1β stimulation increases HIF-1α expression in iTreg

**Figure 2:**
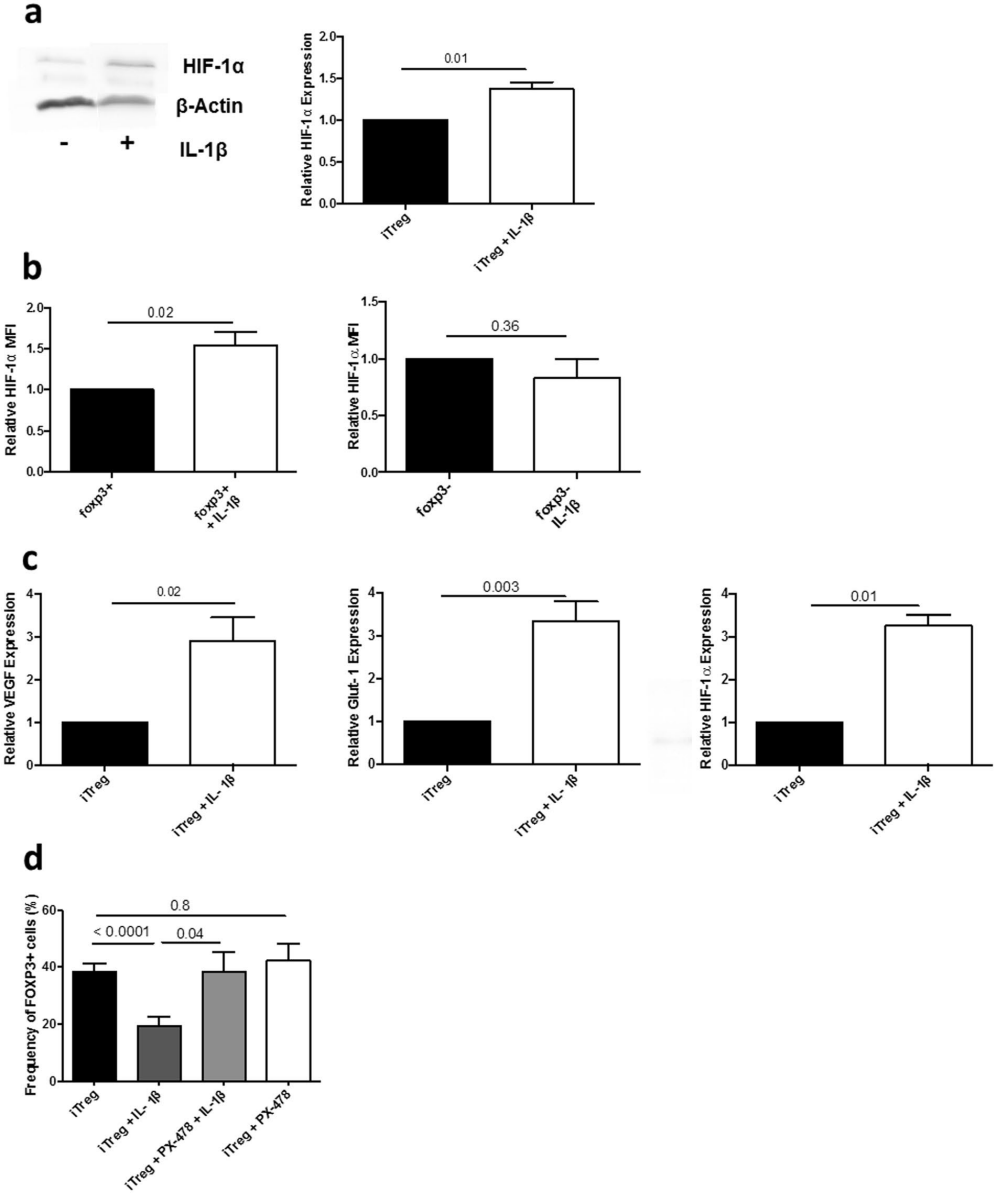
IL-1β stimulation increases HIF-1α expression in iTreg. (**a**) Left panel: HIF-1α expression was analyzed by western blot (WB) at d3 of polarization, with or without IL-1β. Expression of β-actin was used as a loading control in all WB experiments (n = 6). Histograms show the mean (±SEM) fold change in IL-1b treated cells versus untreated iTreg. Right panel: Representative WB experiment. (**b**) HIF-1α expression was also measured by flow cytometry in the FOXP3^+^ and FOXP3^−^ subpopulation at d6 of iTreg polarization, in presence or absence of IL-1β (100 ng/mL). Histograms show the mean (±SEM) fold change in HIF-1α MFI in IL-1β-treated cells in relation to untreated cells (n = 12). (**c**) Relative mRNA levels of VEGF, Glut-1 and HIF-1α of iTreg at d3, in presence and absence of IL-1β (100 ng/mL) (n = 3–6). Expression levels were first normalized to the corresponding 18S expression, and then expression in unstimulated iTreg was assigned the arbitrary value of 1. (**d**) Mean (±SEM) frequency of FOXP3^+^ cells at d6 of iTreg polarization, in presence or absence of IL-1β and the HIF-1α inhibitor PX-478 (10 μM) (n = 12). P values correspond to paired t-tests.

IL-1β had been shown to stabilize HIF-1α in non-hematopoietic cells^8–11^; we thus investigated the effect of IL-1β on HIF-1α expression in iTreg. A stable HIF-1α signal was detectable by western-blot at d3 upon IL-1β stimulation (Fig. 2a). HIF-1α expression in iTreg was further quantified by flow cytometry at d6. IL-1β increased HIF-1α MFI in the FOXP3^+^ population, but not in the FOXP3^−^ population (Fig. 2b). In contrast, IL-1β did not change HIF-1α in T cells cultured for 6 days in un-polarized conditions (data not shown), similar to its lack of effect in FOXP3^−^ cells in Treg-polarizing conditions. It also did not change FOXP3 MFI and had no impact on the expression of HIF-1β (data not shown). To analyze the effect of IL-1β on HIF-1α pathway, we also measured mRNA levels of HIF-1α target genes. VEGF and Glut-1 mRNA levels were up-regulated after IL-1β stimulation at d3 of iTreg polarization (Fig. 2c). In addition, we show that IL-1β transcriptionally induces HIF-1α, as HIF-1α mRNA levels were also increased at d3 (Fig. 2c).

Next, to determine the involvement of HIF-1α induction on the inhibitory effect of IL-1β on iTreg differentiation, we differentiated iTreg in presence of IL-1β and the HIF-1α inhibitor, PX-478^30^. Inhibition of HIF-1α abrogated the effect of IL-1β and restored the percentage of FOXP3^+^ cells to the level seen in untreated iTreg (Fig. 2d). Importantly, PX-478 specifically blocked the effect of IL-1β as it did not alter FOXP3^+^ cell frequency by itself (Fig. 2d).

### HIF-1α induction by IL-1β is mTOR-dependent

**Figure 3:**
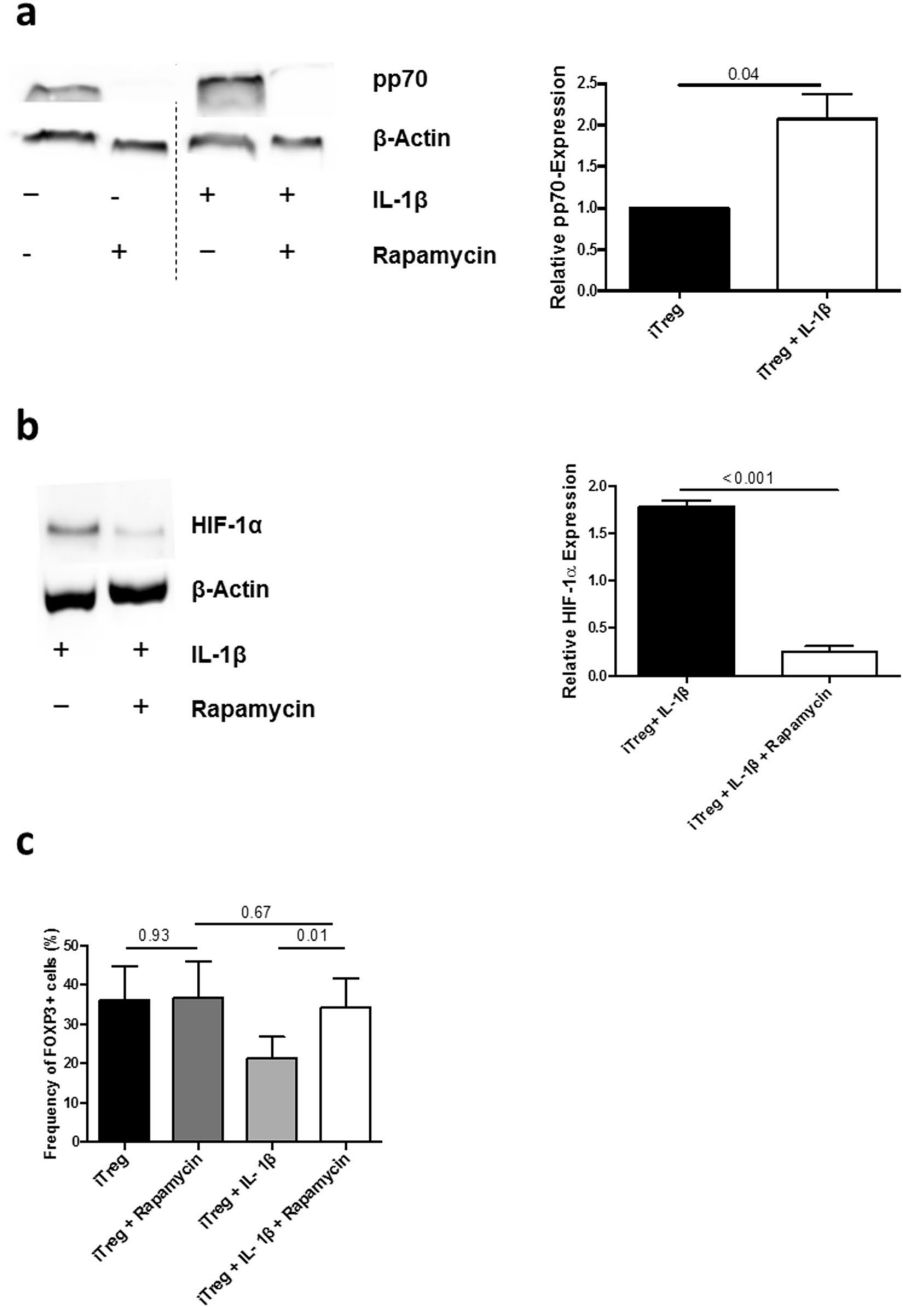
HIF-1α induction by IL-1β is mTORC1 dependent. (**a**) Left panel: representative WB analysis of pp70 expression at d3 in iTreg polarized in presence or absence of IL-1β or rapamycin (25 μM). Right panel: mean (±SEM) fold change in pp70 expression at d3 (n = 6). (**b**) Left panel: representative WB analysis of HIF-1α expression at d3 in iTreg polarized in presence of IL-1β with and without rapamycin (25 μM). Right panel: mean (±SEM) HIF-1α MFI in iTreg differentiated in presence of IL-1β, with or without rapamycin (measured at d3, n = 6). (**c**) Mean (±SEM) iTreg frequency in presence or absence of IL-1β and rapamycin (n = 6). P values correspond to paired t-tests.

The mTORC1 pathway mediates HIF-1α induction in Th17 cells^10^. We could detect enhanced mTORC1 activation in IL-1β-exposed iTreg, as evidenced by higher levels of pp70 (Fig. 3a). In addition, mTOR inhibition by rapamycin suppressed the effect of IL-1β on HIF-1α expression (Fig. 3b). In line with the importance of mTOR in IL-1β-induced HIF-1α, rapamycin abolished the effect of IL-1β on iTreg polarization (Fig. 3c). Rapamycin alone did not alter the frequency of FOXP3^+^ cells (Fig. 3c).

### HIF-1α induction by DMOG abrogates iTreg differentiation

**Figure 4:**
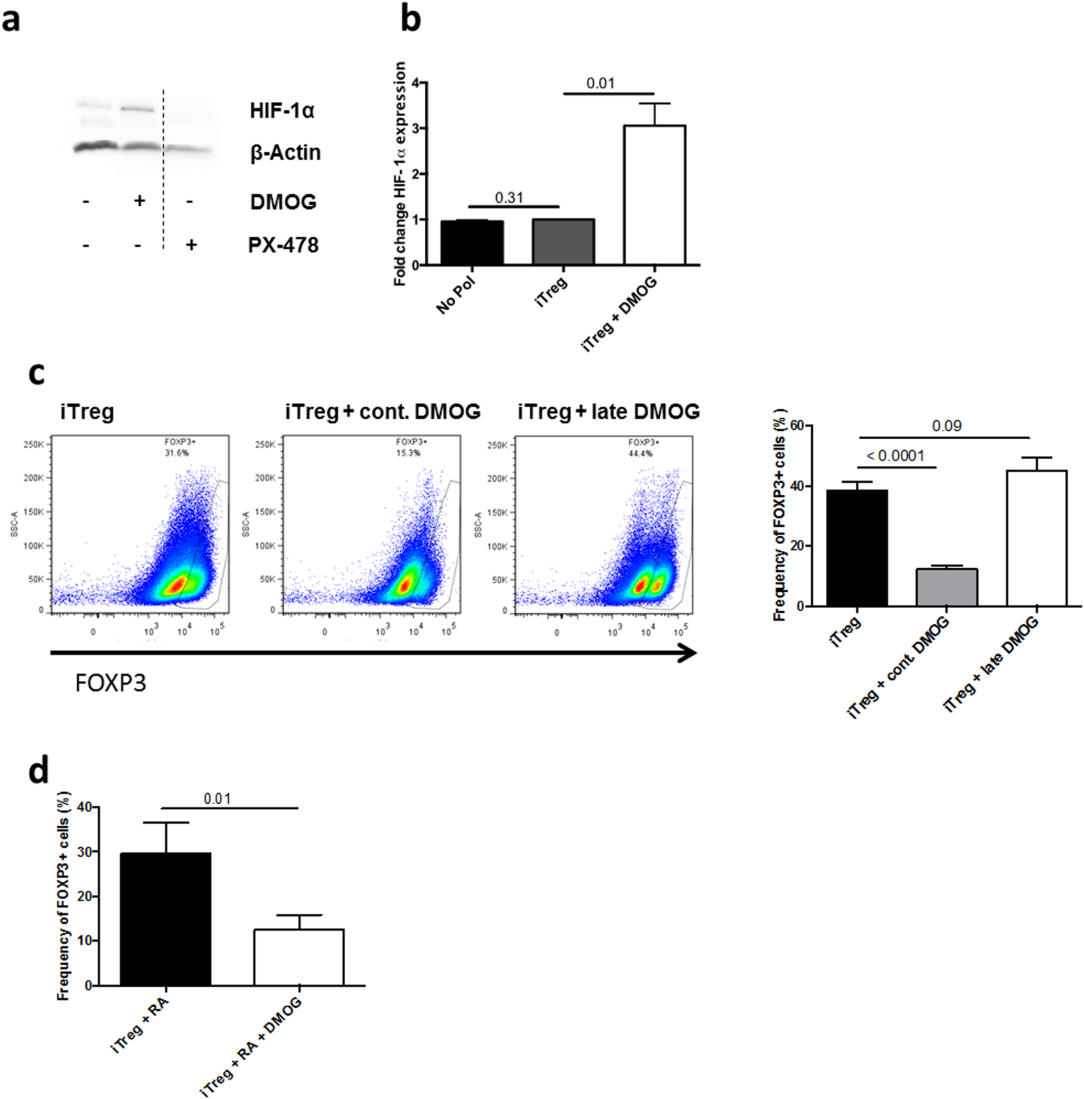
HIF-1α induction by DMOG abrogates iTreg differentiation. (**a**) Representative WB analysis of HIF-1α expression at d3 of Treg polarization in presence or absence of DMOG (100 μM) and PX-478 (10 μM). (**b**) Mean (±SEM) fold change in WB HIF-1α expression in unpolarized CD4^+^ cells (unpol) or iTreg, cultured in absence or presence of continuous DMOG (added every 2 days for 6 days). (**c**) Left Panel: representative FACS dot plots of FOXP3^+^ CD4^+^ cells at d6 of iTreg polarization, with and without DMOG. Right panel: mean (±SEM) frequency of FOXP3^+^ cells in presence or absence of DMOG (n = 12). DMOG was either continuously present (cont. DMOG) or added 3 days after culture initiation (late DMOG). (**d**) Mean (±SEM) iTreg frequency in cells polarized in presence of retinoic acid (RA) (100 nM), in absence or presence of continuous DMOG (n = 6). P-values correspond to paired t-tests.

We next determined whether other pathways that up-regulate HIF-1α expression have similar effects on differentiating iTreg. DMOG, an inhibitor of prolyl hydroxylases, enhanced HIF-1α but not HIF-1β (data not shown) expression in iTreg by 2-fold at d3 (Fig. 4a,b), consistent with its reported stabilization of HIF-1α^31^. Increased HIF-1α expression was evident in both FOXP3^+^ and FOXP3^−^ cells (data not shown). Up-regulation of HIF-1α expression occurred only during continuous presence of DMOG throughout the differentiation, as late addition of DMOG (3 days after culture initiation) had no effect on HIF-1α expression (data not shown). Similar to IL-1β, DMOG had no effect on T cell viability or cell cycle progression, in either Treg-polarizing or unpolarizing conditions (Figure S2).

Consistent with the pattern of HIF-1α upregulation, continuous DMOG treatment significantly reduced the frequency of FOXP3^+^ cells from approximately 40% to 10% (Fig. 4c). Its late addition did not impair iTreg polarization (Fig. 4c). Because retinoic acid (RA) has been shown to improve the stability of FOXP3 expression in human iTreg cells^32, 33^, we tested if HIF-1α upregulation would still affect iTreg differentiation in these conditions. As shown in Fig. 4d, treatment by DMOG still significantly decreased the percentage of FOXP3^+^ cells in these more stringent conditions.

Next, we analyzed whether enhanced HIF-1α expression due to either IL-1β signaling or chemical stabilization changed the differentiation program from Treg to another T cell subset. To do so, we characterized the cytokine expression in the T cells exposed to IL-1β or DMOG during iTreg differentiation, analyzing both the FOXP3^+^ and FOXP3^−^ populations. IL-1β and DMOG did not induce Th2 or Th17 polarization instead of Treg differentiation, as the expression of TNF-α, IL-5 or IL-17 in the overall population was unchanged by these treatments (Figure S3a,b,c). This was also true when we analyzed cytokine expression in either the FOXP3^+^ or FOXP3^−^ subset (data not shown). However, IL-1β treatment increased intracellular IFN-γ expression detected in total CD4^+^ T cells (Figure S3d). These results were confirmed by measuring IFN-γ levels in the d6 supernatants (Figure S3e). However, addition of IFN-γ receptor 1 blocking antibody (αIFNR) to the cultures stimulated with IL-1β did not restore the frequency of FOXP3^+^ cells (Figure S3f), although it decreased IFN-γ levels (data not shown).

### Impact of HIF-1α induction on iTreg mitochondrial respiration and glycolysis

**Figure 5:**
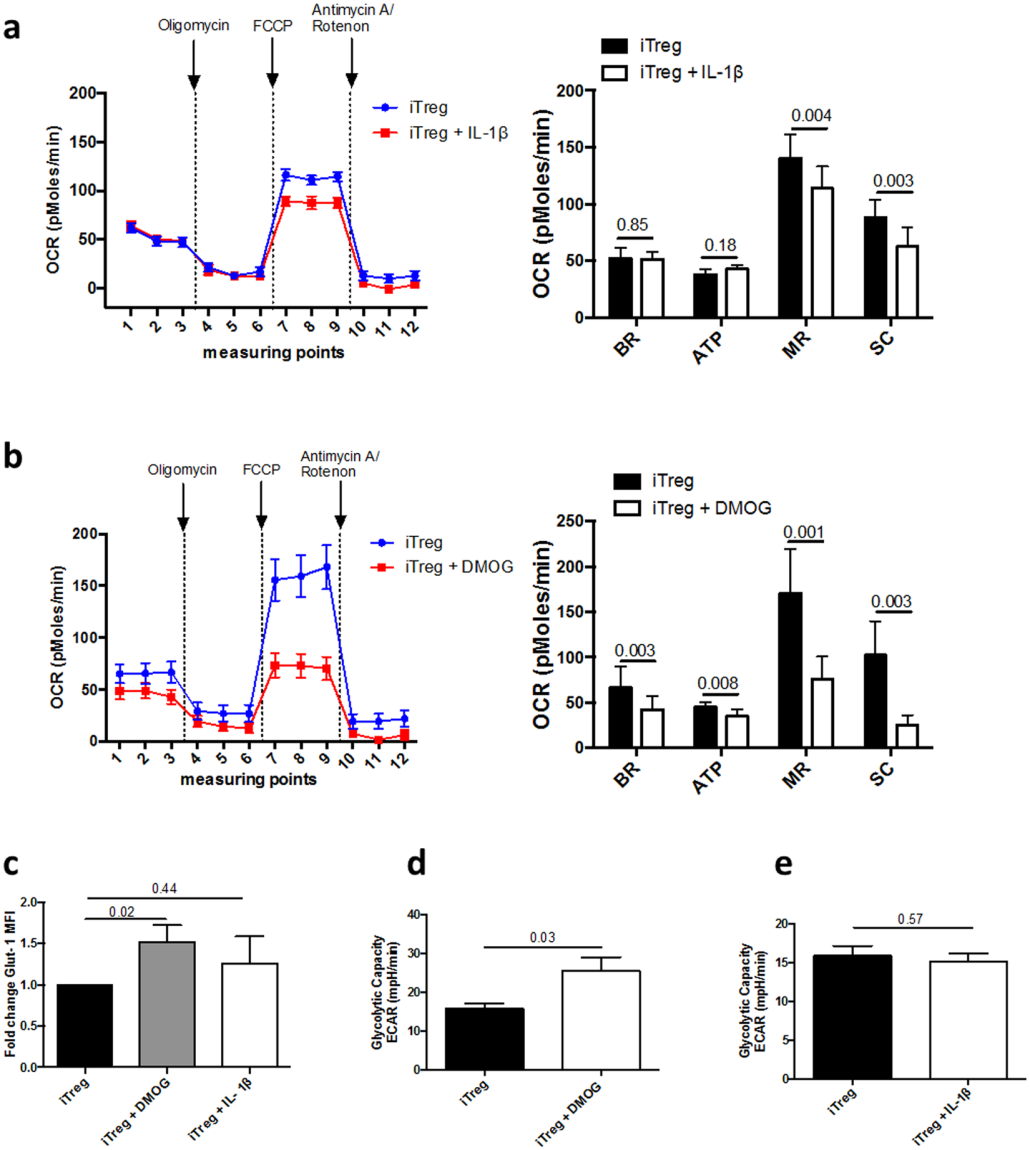
Impact of HIF-1α induction on iTreg mitochondrial respiration. (**a**,**b**) Oxygen consumption rate (OCR) was measured in iTreg differentiated for 2 days in presence of IL-1β (**a**) and 1 day in presence of DMOG (**b**) using a Seahorse instrument and the XF mito stress test kit. Basal respiration (BR) is the OCR measured before adding any additional compound. ATP production (ATP) is estimated based on the difference between basal OCR and OCR after oligomycin treatment. Maximal respiration (MR) is defined as the OCR after FCCP treatment. Spare capacity (SC) is the difference between maximal and basal respiration. Data of 3 independent experiments. (**c**) Mean (±SEM) Glut-1 expression measured by flow cytometry in iTreg, untreated or treated with IL-1β or DMOG (n = 12). (**d**,**e**) Extracellular acidification rate (ECAR) was measured by seahorse in iTreg untreated or and treated with (**d**) DMOG or (**e**) IL-1β. Mean (±SEM) values of 3 independent experiments are shown. P values correspond to paired t-tests.

We next analyzed iTreg mitochondrial respiration in the early phase of differentiation in presence or absence of IL-1β or DMOG. Interestingly, IL-1β, although it stabilized HIF-1α, had only a minor effect on mitochondrial respiration, affecting only maximal respiration and spare capacity (Fig. 5a). In contrast, DMOG decreased the basal respiration, ATP production, maximal respiration and spare capacity of iTreg, indicating a decreased mitochondrial respiration (Fig. 5b). We also measured the expression pattern of the HIF-1 dependent Glucose transporter-1 (Glut-1) and the glycolytic capacity of iTreg upon IL-1β and DMOG stimulation. Again, DMOG stimulation, but not IL-1β, increased the Glut-1 expression at d6 of polarization (Fig. 5c). Accordingly, DMOG, but not IL-1β, increased iTreg glycolytic capacity (Fig. 5d,e).

To further investigate the importance of metabolic changes in the HIF-1α dependent inhibition of iTreg polarization, we analyzed the effect of FAO-inhibition by Etoxomir (Etx) on human iTreg polarization and HIF-1α expression. Etx significantly increased HIF-1α expression in the FOXP3^+^ population, but not the FOXP3^−^ cells cultured in Treg-polarizing conditions (Figure S4a). Similar to the effect seen in IL-1β and DMOG-exposed cultures, Etx treatment profoundly inhibited iTreg polarization (Figure S4b). Etx treatment by itself did not alter iTreg or Tcon viability or cell cycle progression (Figure S2). However, the combination of Etx and PX-478 severely diminished cell viability. Therefore, we could not determine whether the effect of Etx on iTreg differentiation was mediated by its induction of HIF-1α expression. In contrast to IL-1β, Etx stimulation did not increase mTORC1 expression and accordingly, rapamycin did not inhibit the effect of Etx on iTreg polarization (data not shown). Similar to DMOG, Etx treatment reduced iTreg mitochondrial respiration (Figure S4c).

## Discussion and Conclusion

Uncontrolled immune responses are key factors in the etiology of many autoimmune diseases and chronic inflammatory processes. As Tregs are essential to control both adaptive and innate immune responses, understanding the mechanisms that govern human Treg differentiation is crucial for improving therapeutic strategies.

IL-1β is needed for Th17 differentiation^34, 35^, and consequently is a critical pathogenic mediator in many autoimmune diseases. Herein, we show for the first time that IL-1β also abrogates iTreg generation, which would reinforce its pathogenic role. Moreover, we show that IL-1β acts through mTORC1 activation, as its effect was blocked by rapamycin, which acts mainly on mTORC1, while having only minor effects on mTORC2^36^. These data confirm the negative role exerted by mTOR on iTreg differentiation, which had been suggested by the enhanced iTreg generation occurring in mice with a mTORC1 deletion^37^. Rapamycin has been described to enhance Treg differentiation, in absence of inflammatory stimuli, but increased Treg survival is considered to be the principal underlying mechanism^38, 39^. Our data suggest that blocking mTORC1 would also be very beneficial in a context where inflammation is present, to prevent the deleterious effect of IL-1R signaling on Treg differentiation. We also show that, downstream of mTOR activation, IL-1β signaling inhibits Treg differentiation through HIF-1α induction at the transcriptional level, as an HIF-1α inhibitor reversed IL-1β deleterious effect. The induction of HIF-1α by IL-1β was previously described, with either the direct activation of HIF-1α transcription by CCAAT/enhancer-binding protein δ^40^, or the activation of the NF-κB and PI-3kinase-AKT-mTOR signaling pathway evoked as underlying mechanisms^9^. The role of HIF-1α was further ascertained by the fact that its stabilization by DMOG also abrogated Treg differentiation, as profoundly as IL-1β did. Our data thus precisely map the signaling pathways that can inhibit human Treg differentiation in an inflammatory context. We did not observe any impact on the expression of HIF-1β. Of note, we focused on the effect of IL-1β since it is one of the most important pro-inflammatory cytokines, but it is likely that exposure to IL-6 or TNF-α would have similar consequences, as these other pro-inflammatory cytokines also induce HIF-1α in non-hematopoietic cells and murine T cells^8, 10^.

Surprisingly because HIF-1α was reported to control the balance of Th17/Treg differentiation^10, 18, 41^, IL-1β and DMOG did not prime CD4^+^ T cells towards a Th17 differentiation profile. It should be noted that this effect of HIF-1α on IL-17 production was previously seen in the context of HIF-1α knockout models or in presence of IL-23-producing antigen presenting cells (APC), which maintains IL-17 production^42–44^. In our experimental system devoid of APC, IL-17 production might have been too transient to be detected. IL-1β increased modestly (less than 2-fold), IFN-γ expression, similar to what was reported in human activated T cells^44^, but this mechanism did not significantly contribute to the inhibition of FOXP3 expression. IFN-γ was shown to inhibit iTreg polarization^45^, but at much higher doses (10 ng/ml) than the levels we measured in IL-1β-treated cultures (<1 ng/ml). In contrast to IL-1β, DMOG treatment decreased the percentage of IFN-γ^+^ cells, which is consistent with the inhibitory effect of HIF-1α overexpression on IFN-γ expression^46^. The fact that DMOG decreased the frequency of IFN-γ^+^ cells also suggests that IL-1β-mediated induction of IFN-γ is independent of its effect on HIF-1α.

Metabolism profoundly affects Treg polarization. Notably, enhanced glycolysis or FAO inhibition prevents murine iTreg differentiation^10, 27, 47^. The inhibition of glycolysis, for example by using 2-Deoxy-D-Glucose (2-DG), promotes iTreg polarization by regulating mTOR activity^47^, which could be linked to its downregulation of HIF-1α expression, as described in tumor cell lines^48, 49^. As previous studies described HIF-1α as an important regulator of cell metabolism^10, 18, 50^, we thus analyzed the effect of IL-1β and DMOG on Treg metabolism. Surprisingly, in our study, the induction of HIF-1α by IL-1β had only minor effects on iTreg mitochondrial respiration and glycolysis. These moderate effects might nevertheless have been sufficient to inhibit the differentiation in iTreg, in addition to the induction of the mTORC1 pathway. Alternatively, direct binding of HIF-1α to FOXP3 and subsequent proteasomal degradation, could be the major underlying mechanism, as has been proposed in several murine models^10, 51^. DMOG had a more profound effect on iTreg metabolism, clearly inhibiting mitochondrial respiration. However, the kinetic of HIF-1α upregulation versus that of metabolic changes in DMOG-treated cells should be kept in mind while interpreting these data. In our study, metabolic changes preceded HIF-1α stabilization in cells exposed to DMOG, as they were already apparent at d1, whereas constant HIF-1α stabilization was detectable only at d3. Therefore, it is possible that DMOG early inhibition of iTreg mitochondrial respiration, similar to what has been described in cancer cell lines^31^, might be essential in diminished iTreg polarization, in addition to HIF-1α-dependent mechanisms.

Taken together, our data show that exposure to inflammatory stimuli profoundly inhibits human Treg differentiation, and that HIF-1α upregulation is essential in this process. However, this deleterious effect of inflammatory cytokines does not appear to be completely driven by metabolic changes, contrarily to what was previously reported concerning HIF-1a function. These data thus suggest that several mechanisms contribute to the regulation of iTreg differentiation, but the timing and respective requirement for each pathway vary depending on the milieu in which iTreg differentiate. Our data also suggest that targeting HIF-1α could be a strategy to foster iTreg differentiation in an inflammatory milieu. However, the dynamics of HIF-1α stabilization, and downstream consequences, will need to be further elucidated *in vivo*.

## Materials and Methods

### Regulatory and conventional T cell purification

PBMC were isolated with Ficoll-Hypaque gradients (GE Healthcare, Fairfield, CT) from healthy adults recruited at the Hoxworth Blood Center (Cincinnati, OH, USA) and in Luebeck (Department of Infectious Diseases and Microbiology, Germany). Resting CD4^+^ T cells were purified by negative selection using magnetic beads, according to the manufacturer’s instructions (Miltenyi Biotec, Auburn, CA). Purified CD4^+^ T cells were stained with anti-CD8 FITC (RPT-T8), anti-CD25 APC (M-A251) (both﻿ BD Biosciences, San Jose, CA), and anti–CD127 PE (eBioRDR5; Beckman Coulter, Fullerton, CA). Tcons defined as CD4^+^CD25^−^CD127^high^ were sorted using Cell Sorter FACS Aria III (BD Bioscience). In addition, circulating regulatory T cells were sorted based on their CD4^+^CD25^+^CD127^Low/−^ phenotype (resulting in cells >90% FOXP3+).

### iTreg polarization

Purified Tcons were stained with 0.5 µM cellTracker^TM^ violet (Invitrogen, Grand Island, NY). Tcons were activated in a 24-well tissue culture plate for 6 days at 37 °C and 5% CO_2_, using anti–CD3/anti-CD28 beads (Invitrogen) at a 1:1 bead-cell ratio, in X-VIVO15 medium (Lonza, Basel, Switzerland) and 100 U/ml IL-2 (provided by the National Institutes of Health AIDS Research and Reference Reagent Program, Bethesda, MD). iTreg polarization was performed in the presence of 1 ng/ml of TGF-β (Miltenyi Biotec); in some experiments, 100 nM of RA (Sigma-Aldrich, St. Louis MO) was added to the culture. Cytokines were replaced at d3, in the same concentrations. After 6 days, the cultures were stimulated for 4 h with 10 ng/ml PMA (Sigma-Aldrich) and 1 μg/ml ionomycin (Calbiochem, San Diego, CA), in presence of brefeldin A (10 µg/mL, Sigma-Aldrich) and monensin (1:1000 dilution; eBioscience, San Diego CA).

### Inhibition of iTreg polarization

Purified Tcons were cultured in iTreg polarizing conditions in the presence of 100 ng/ml of recombinant human IL-1β (Peprotech, Rocky Hill, NJ) or 100 μM DMOG, an inhibitor of prolyl hydroxylases (Cayman Chemical, Ann Arbor, MI). In some experiments, HIF-1α stabilization was inhibited by 10 μM PX-478 (MedKoo Biosciences, NC). PX-478 and DMOG were added every other day. mTOR was inhibited with 25 μM of rapamycin (Sigma-Aldrich), added at d1 and d3. Anti-IFN-γ R1 blocking antibody (Clone 92101, R&D Systems﻿, Boston MA) was added at days 0, 3 and 4.

### Flow cytometry assays

Cells were treated with human IgG to block Fc-receptors and stained for surface markers. Cells were then washed and fixed, followed by permeabilization according to the manufacturer’s instructions (eBioscience). Cells were finally stained for intracellular markers and analyzed with a FACS LSR-II (BD Biosciences). Flow cytometry analysis was performed using FACSDiva software version 6.1.2 (BD Biosciences). The dyes and human antibodies included in the flow cytometry panel were: CellTracker^TM^ violet, LIVE/DEAD® aqua Fixable Dead Cell Stain kit (Invitrogen); anti-Ki67-PerCP-Cy5.5 (B56, BD Biosciences); anti-FOXP3-eF660 (PCH101), anti-IL-17PE (eBio64DEC17) (eBioscience); anti-Glut-1-AF700 (202915, R&D Systems). In addition 0,5 µg HIF-α polyclonal antibody (Cayman Chemical) was labeled with Zenon AF488 (Thermo Fisher Scientific, Rockford, IL). In another flow cytometry panel, production of cytokines was determined using CellTracker^TM^ violet, LIVE/DEAD aqua, anti-IL2-FITC (5344.111), anti-IL5-APC (TRFK5), anti-IFNγ-PerCP-Cy5.5 (B27), anti-TNFα-PeCY7 (Mab11) (all ﻿BD Biosciences), anti-FOXP3-PE and anti-IL-17-APC-eF780 (eBioscience). Live cells were gated based on forward- and side-scatter properties and the absence of LIVE/DEAD stain. Doublets were excluded. To set up the positive gate for FOXP3, we used unpolarized cells as negative population. In addition, appropriate isotype controls were used for each antibody.

### Measurement of mitochondrial respiration

Oxygen consumption rate (OCR) was measured with XFe24 Extracellular Flux Analyzer (Seahorse Bioscience Copenhagen, Denmark). Purified Tcons were activated with anti-CD3/anti-CD28 Dyanbeads for 24 h in Seahorse culture microplates. Cell adherence was ensured by coating the culture microplates with Cell tak (BD Bioscience) following the manufacturer’s instructions. After 24 h, the medium was changed to XF Assay Medium (350 μL per well, pH = 7.4) and equilibrated for 1 h at 37 °C without CO_2_, before the mitochondrial stress test was started (XF mito stress test kit, Seahorse Bioscience). Oligomycin (1 μM), FCCP (0.9 μM) and antimycin A/rotenone (1 μM) were successively injected while measuring OCR. Basal respiration was defined as baseline OCR. Maximal respiration was defined as OCR after FCCP treatment. ATP production was calculated as the difference between basal respiration and OCR after oligomycin treatment, and spare capacity was calculated as the difference between maximal and basal respiration.

### Measurement of extracellular acidification rate

Extracellular acidification rate (ECAR) was measured using the XFe24 Extracellular Flux Analyzer and the glycolysis stress test kit (Seahorse Bioscience). Cells were prepared as described above, with the difference that the medium was changed to a medium without Glucose (pH = 7.35). Glucose (2 mM), oligomycin (1 μM) and 2-desoxy-D-glucose (2 μM) were then successively added while measuring ECAR. Glycolytic capacity was defined as ECAR after glucose and oligomycin injection.

### Western Blot analysis

The cells were lysed in lysis buffer (125 mM Tris-HCl pH = 7.8, 20% glycerol, 4% SDS, 0.1 M dithiothreitol, bromphenol blue (Sigma - Aldrich). Protein separation was performed on 4% polyacrylamide gel and electro blotted on nitrocellulose membrane (Whatman Inc., Florham Park, NJ.) TBS (0.1% Tween)/5% fat-free milk was used for membrane blockade. Membranes were incubated with anti-HIF-1α-antibody or anti-pp70-antibody at 4 °C overnight, followed by horseradish peroxidase- linked anti-mouse IgG antibody (both from Cell Signaling Technologies, Danvers, MA), and enhanced chemiluminescence substrate (Thermo Fisher Scientific), before analysis by an automated image acquisition system (Fusion FX7, Vilber Lourmat, Eberhardzell, Germany). Protein amounts were quantified using Bio1D software (Vilber Lourmat). Anti-β-actin (Cell Signaling Technologies) served as loading and blotting control in all experiments. In case additional samples are displayed on the initial blots that are not relevant to this study, the images were cropped without additional modifications before analysis. Full-length blots are included in Figure S5.

### Analysis of mRNA expression

Cells were lysed and total RNA was extracted utilizing NucleoSpin RNA 2 Kit (Machery-Nagel, Düren, Germany). RNA was transcribed into cDNA using First-Strand PCR Kit (Roche, Basel, Switzerland). qRT-PCR was performed with Light Cycler Detection System, using primers from TIB Molbiol (Berlin, Germany)﻿. Relative mRNA expression of HIF-1α (forward: 5′-GGCAGCAACGACACAGAAACTGA, reverse: 5′-TGATCCTGAATCTGGGGCATGGT), Glut-1 (forward: 5′-GGTTGTGCCATACTCATGACC, reverse: 5′-CAGATAGGACATCC AGGGTAGC), and VEGF (forward: 5′-CCTTGCTGCTCTACCTCCAC, reverse: 5′-CACACAGGATGGCTTGAAGA) was performed against 18S rRNA (forward: 5′-TCAAGAACGAAAGTCGGAGG, reverse: 5′-GGACATCTAAGGGCATCACA).

### Data analysis

Comparison of untreated and treated conditions was performed by paired t-tests using GraphPad Prism, version 5.0 for Windows (La Jolla, CA). P-values ≤ 0.05 were considered as indicative of significant differences.

## Electronic supplementary material

Supplementary Information
