# Supplementary material for: IL-1β induced HIF-1α inhibits the differentiation of human FOXP3+ T cells
Source: Sci Rep. 2017 Mar 28;7:465. doi: 10.1038/s41598-017-00508-x (PMC5428734; doi:10.1038/s41598-017-00508-x)
Supplement: Supplementary file 1 — Supplementary Information [file 41598_2017_508_MOESM1_ESM.pdf]

Supplementary File

IL-1β induced HIF-1 α inhibits the differentiation of human FOXP3$^+$ T cells

Lea M. Feldhoff[1], Cesar M. Rueda[2], Maria E. Moreno-Fernandez[2], Johanna Sauer[1], Courtney M. Jackson[2], Claire A. Chougnet[2¶], Jan Rupp[1¶*]

[1] Department of Infectious Diseases and Microbiology, University of Lübeck, Lübeck, Germany.

[2] Division of Immunobiology, Cincinnati Children's Hospital Research Foundation, Department of Pediatrics, University of Cincinnati College of Medicine, Cincinnati, Ohio. USA.

Supplementary Figures

Figure S1

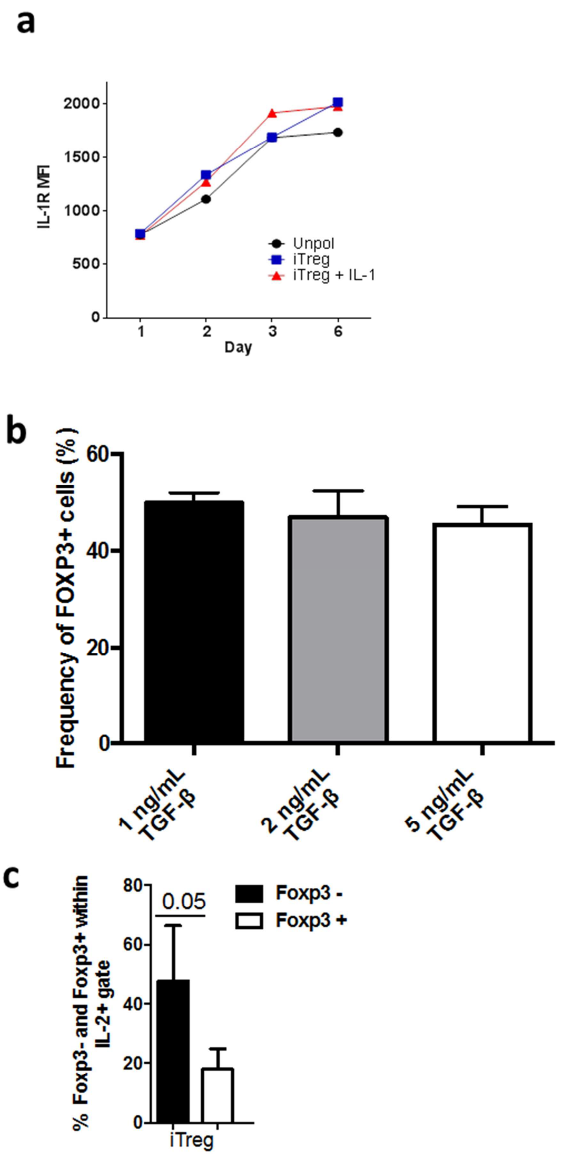

Figure S1. a) Kinetics of IL-1β receptor (IL-1R) expression (Mean of 6 experiments. IL-1R expression was measured by flow cytometry in $CD4^{+}FOXP3^{-}$ T cells cultured in unpolarized (unpol) or Treg-skewing conditions (iTreg) in absence or presence of IL-1β (n=6). b) Frequency of $FOXP3^{+}$ cells analyzed by FACS at d6 of iTreg polarization using different TGF-β concentrations (1, 2 or 5 ng/mL) (n=3). c) Frequency of IL-2^{+} cells in the $FOXP3^{+}$ and $FOXP3^{-}$ subpopulation at d6. Cells were restimulated with PMA/Ionomycin for 5h (n=12).

Figure S2

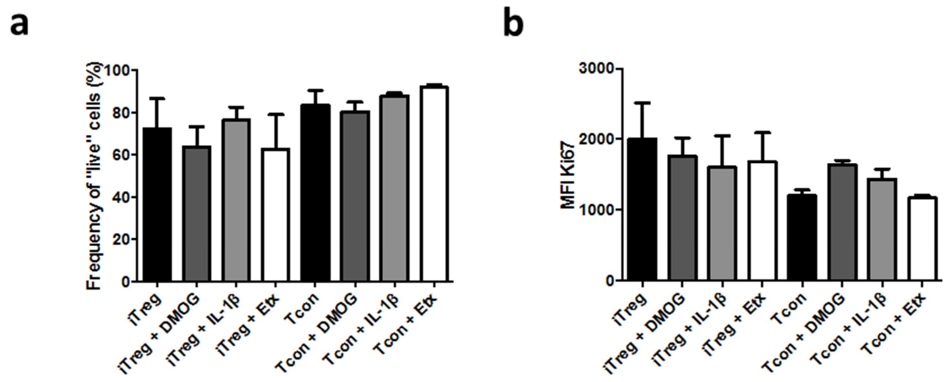

Figure S2. Mean (±SEM) percentage of live cells (a) and Ki67 MFI (b) in iTreg or Tcon. Cells were cultured for 6 days in absence or presence of IL-1β, DMOG or Etoxomir (Etx). Cells were stained with the LIVE/DEAD® Stain kit and intracellularly with anti-Ki67 and analyzed by flow cytometry (n= 8).

Figure S3

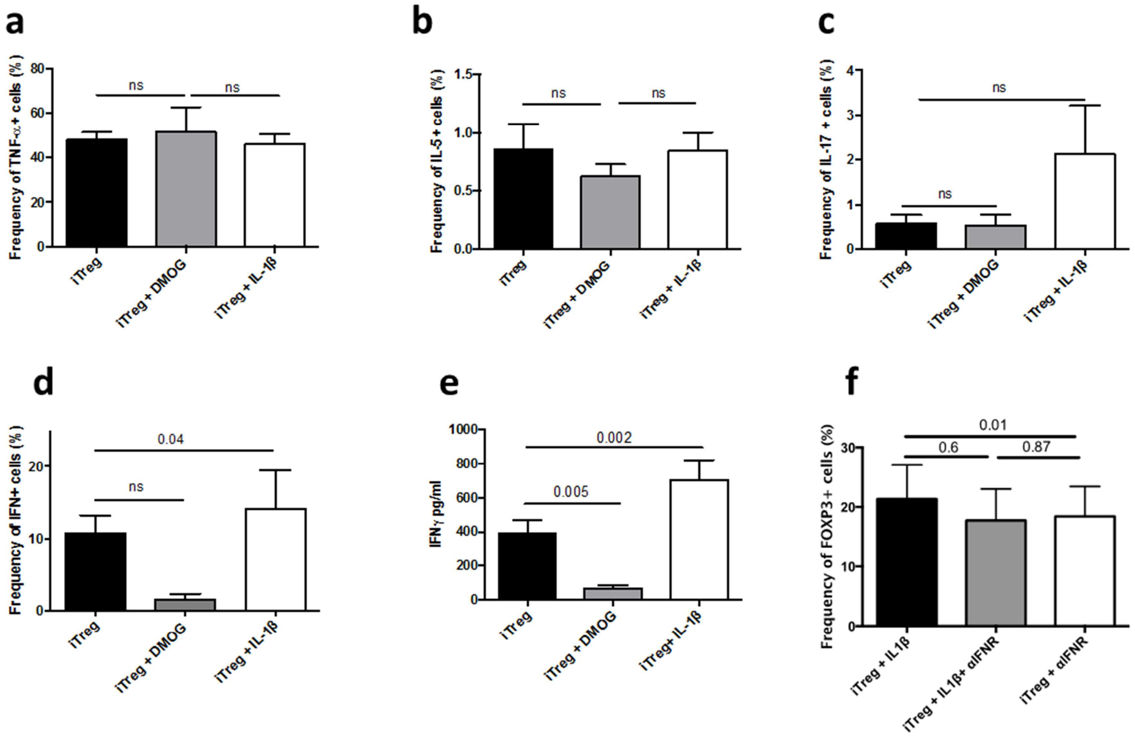

Figure S3. a-d) Flow cytometry analyses of intracellular expression of TNF-α, IL-5, IL-17 and IFNγ in cells analyzed at d6 of iTreg polarization. iTreg were untreated or treated with DMOG or IL-1β and analyzed after a brief restimulation by PMA/Ionomycin. Mean (±SEM) are shown (n=6). e) Mean (±SEM) IFNγ levels were analyzed by ELISA in the d6 culture supernatants (n=6). f) Mean (±SEM) frequency of FOXP3+ cells in IL-1β-exposed cultures in presence or absence of blocking anti-IFNγ receptor antibody (n=6). P values correspond to paired t-tests.

Figure S4

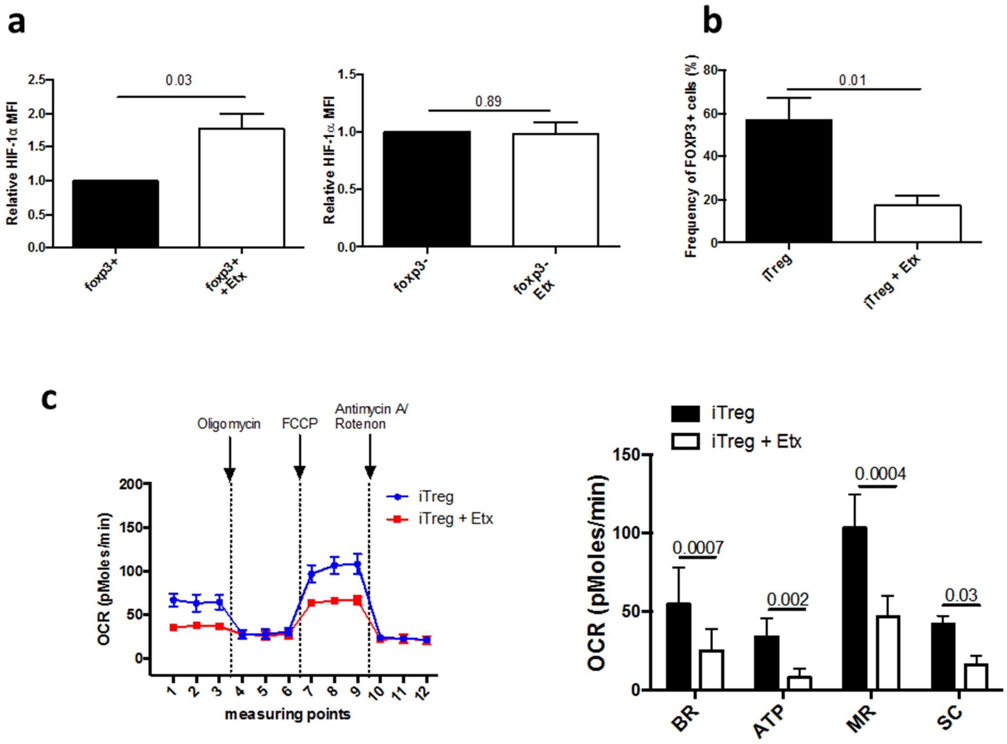

Figure S4. a) Fold change of HIF-1α expression, measured by flow cytometry in the FOXP3⁺ and FOXP3⁻ subpopulation at d6 of iTreg polarization in presence or absence of Etx (200 µM) (n=6). b) Mean (+SEM) iTreg frequency at d6 in CD4+ T cells cultured in Treg-polarizing conditions in presence or absence of Etx (n=6). c) Oxygen consumption rate (OCR) was measured in iTreg differentiated for 1 day in presence of Etx using a Seahorse instrument and the XF mito stress test kit. Basal respiration (BR) is the OCR measured before adding any additional compounds. ATP production (ATP) is estimated based on the difference between basal OCR and OCR after oligomycin treatment. Maximal respiration (MR) is defined as the OCR after FCCP treatment. Spare capacity (SC) is the difference between maximal and basal respiration. Mean (+SEM) values of 3 independent experiments. P values correspond to paired t-tests.

Figure S5

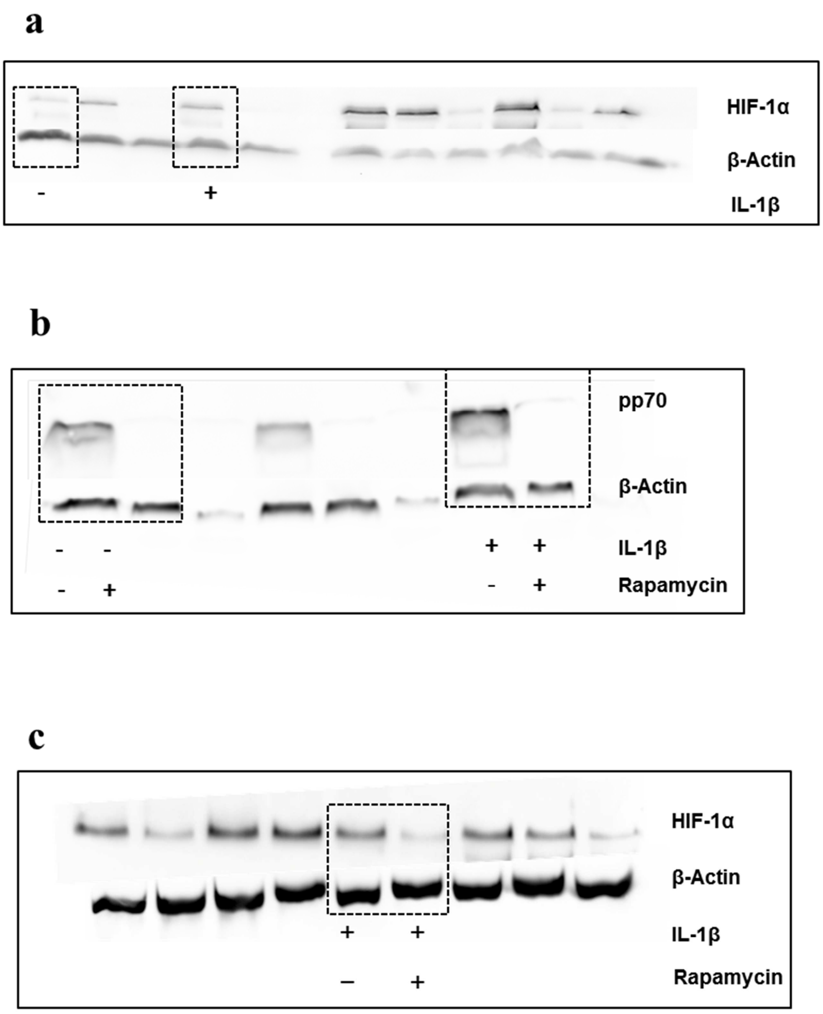

Figure S5. According to the policy of *Science Reports* the full-length blots of the Western Blot analyses shown in Fig. 2a, Fig. 3a,b are included in the Supplementary Information.
